# Next Generation Cytogenetics in Myeloid Hematological Neoplasms: Detection of CNVs and Translocations

**DOI:** 10.3390/cancers13123001

**Published:** 2021-06-15

**Authors:** María Chicano, Diego Carbonell, Julia Suárez-González, Sergio Lois, Mercedes Ballesteros-Culebras, Cristina Andrés-Zayas, Paula Muñiz, Gabriela Rodríguez-Macias, Mariana Bastos-Oreiro, Patricia Font, Mónica Ballesteros, Mi Kwon, Javier Anguita, José Luis Díez-Martín, Ismael Buño, Carolina Martínez-Laperche

**Affiliations:** 1 Department of Hematology, Gregorio Marañón General University Hospital, 28009 Madrid, Spain; maria.chicano@salud.madrid.org (M.C.); diego.carbonell@iisgm.com (D.C.); mariamercedes.ballesteros@salud.madrid.org (M.B.-C.); paula.muniz@iisgm.com (P.M.); gabriela.rodriguez@salud.madrid.org (G.R.-M.); marianabeatriz.bastos@salud.madrid.org (M.B.-O.); patricia.font@salud.madrid.org (P.F.); monica.ballesteros@salud.madrid.org (M.B.); mi.kwon@salud.madrid.org (M.K.); javier.anguita@salud.madrid.org (J.A.); jdiezm@salud.madrid.org (J.L.D.-M.); ismaelbuno@iisgm.com (I.B.); 2 Gregorio Marañón Health Research Institute (IiSGM), 28009 Madrid, Spain; julia.suarez@iisgm.com (J.S.-G.); cristina.andres@iisgm.com (C.A.-Z.); 3 Genomics Unit, Gregorio Marañón General University Hospital, IiSGM, 28009 Madrid, Spain; 4 Sistemas Genómicos, 46980 Valencia, Spain; sergio.lois@sistemasgenomicos.com; 5 Department of Medicine, School of Medicine, Complutense University of Madrid, 28040 Madrid, Spain; 6 Department of Cell Biology, School of Medicine, Complutense University of Madrid, 28040 Madrid, Spain

**Keywords:** cytogenetics, myeloid neoplasms, NGS

## Abstract

**Simple Summary:**

Conventional cytogenetic approaches are the gold standard for the identification of chromosomal alterations in myeloid neoplasms. Next-generation sequencing panels are a new approach for the detection of copy number variations (CNV) or translocations. Here we report on a commercial panel utility including frequent mutations, CNVs and translocations in myeloid neoplasms. A total of 135 patients with myeloid neoplasms and three with acute lymphoblastic leukemia were analyzed by NGS. When comparing with gold standard techniques, 48 frequent alterations were detected by both methodologies, ten of them observed only by conventional methods and another eight only by NGS. Additionally, 38 secondary CNVs were detected in any of the genes included in the panel for mutational analysis. With those results we determine that NGS represents a reliable complementary source of information for the analysis of CNVs and translocations.

**Abstract:**

Conventional cytogenetics are the gold standard for the identification of chromosomal alterations recurrent in myeloid neoplasms. Some next-generation sequencing (NGS) panels are designed for the detection of copy number variations (CNV) or translocations; however, their use is far from being widespread. Here we report on the results of a commercial panel including frequent mutations, CNVs and translocations in myeloid neoplasms. Frequent chromosomal alterations were analyzed by NGS in 135 patients with myeloid neoplasms and three with acute lymphoblastic leukemia. NGS analysis was performed using the enrichment-capture Myeloid Neoplasm-GeneSGKit (Sistemas Genómicos, Spain) gene panel including 35 genes for mutational analysis and frequent CNVs and translocations. NGS results were validated with cytogenetics and/or MLPA when possible. A total of 66 frequent alterations included in NGS panel were detected, 48 of them detected by NGS and cytogenetics. Ten of them were observed only by cytogenetics (mainly trisomy 8), and another eight only by NGS (mainly deletion of 12p). Aside from this, 38 secondary CNVs were detected in any of the genes included mainly for mutational analysis. NGS represents a reliable complementary source of information for the analysis of CNVs and translocations. Moreover, NGS could be a useful tool for the detection of alterations not observed by conventional cytogenetics.

## 1. Introduction

Chromosomal gains and losses as well as specific translocations are common findings in hematological malignancies, usually with a clear impact in diagnosis and prognosis of the disease. Thereby, alterations such -7/7q, -5/5q- or 17p- and rearrangements similar to those involving the *KMT2A* gene, are associated with poor prognosis in myeloid neoplasms (MN) [1,2,3]. Thus, the detection of these alterations is essential to ensure an optimal clinical management of each patient. Karyotyping and fluorescence in situ hybridization (FISH) are the gold standard methods for the detection of chromosomal alterations [4]. The main advantage of karyotyping is the ability to provide a global vision of the status of all chromosomes; however, an optimal cell culture growth is not always available and due to its low-resolution, complementary methods are necessary to achieve thorough results. Furthermore, FISH is useful in those cases where the karyotype is not available and its higher resolution makes it a useful method for the detection of cryptic alterations not observed by karyotype [5]. Array-CGH is also used as a complementary technique in some laboratories for the detection of cryptic copy number variants (CNV) in MN. It allows the identification of large chromosomal alterations all along the genome with a higher resolution than karyotype and FISH [6,7]. However, this kind of technology is not available for all laboratories and it is not able to detect alterations which are present in less than 20% of the cells [8,9,10].

Chromosomal alterations are not the only markers to analyze in order to achieve the best risk stratification for MN patients. With the incorporation of new genetic markers in the updated guidelines for diagnosis and prognosis of myeloid diseases, the number of genes to analyze has increased [2,11]. This fact, together with the emergence of new targeted therapies for the treatment of hematological patients, emphasizes the need for an integrated genetic analysis. Therefore, next-generation sequencing (NGS) has emerged as a method capable of detecting variants in a large number of genes simultaneously, and is being gradually implemented in clinical routine testing. Whole genome/exome sequencing studies for CNV detection have previously been reported [12,13,14]. However, the process of reaching an optimal depth for mutational analysis in neoplastic samples makes them less cost effective for clinical routine testing. In this way, the use of NGS gene panels is a good alternative for the detection of short insertions/deletions and point mutations of clinical relevance in MN. Nevertheless, there are few NGS panels designed to detect large alterations such as CNVs or translocations, most of them being custom panels [15,16,17]. Even so, they use custom algorithms for CNV interpretation, which make it difficult to include this type of methodology in clinical testing. In this context, we report on the detection of frequent CNVs and translocations in MN through a commercial NGS capture-based panel and on the comparison of the results with those obtained by gold standard methodologies.

## 2. Materials and Methods

### 2.1. Patients

The NGS gene panel was performed in 135 patients with confirmed diagnosis of MN and in 3 patients diagnosed with acute lymphoblastic leukemia (ALL) at Gregorio Marañón General University Hospital. A total of 112 consecutive patients between October 2017 and December 2018 were analyzed prospectively: 74 samples were collected at diagnosis, 10 were at relapse samples and 28 were follow-up samples (refractory disease), included for mutational screening as candidates for novel clinical trials. A retrospective analysis included 26 selected patients: 18 at diagnosis, four at relapse and the other 4 were follow-up samples. Among the whole cohort of 138 patients, 49 had acute myeloid leukemia (AML), 38 myelodysplastic syndromes (MDS), 33 myeloproliferative neoplasms (MPN), 10 myelodisplastic/myeloproliferative neoplasms (MDS/MPN) and 5 acute leukemia of ambiguous lineage (MPAL) (Figure S1). Also, three patients with ALL were exceptionally included in order to screen the mutational status in these pathologies of some reported myeloid genes (*IKZF1*, *ETV6*, *FLT3*, *KRAS*, *NRAS*, *DNMT3A*, *TP53*) [18,19]. The study was approved by the local ethics committee (No. 03/201503, 3 March 2015) and all patients provided signed informed consent under the guidelines of the Declaration of Helsinki.

### 2.2. NGS Analysis

Bone marrow sample was used in 118 patients and peripheral blood in those cases where no BM sample was available (20 patients).

Genomic DNA was extracted following the manufacturer’s instructions (Maxwell^®^ 16 Blood DNA Purification Kit; Promega, Madison, WI, USA). NGS analysis was performed using the commercial enrichment-capture gene panel Myeloid Neoplasm-GeneSGKit (Sistemas Genómicos, Valencia, Spain), with a target size of 3 Mb, which includes the detection of variants and CNVs in 35 genes and most frequent CNVs and translocations in MN. A total of 35 genes were included for variants and SNPs/indels screening; additionally, 18 genes were included for CNVs and 14 for rearrangement analysis (Table S1).

Paired-end sequencing (2 × 101 bp) was performed using the Illumina MiSeq platform (Illumina, San Diego, CA, USA). FASTQ files were aligned against the human reference genome (version GRCh38/hg38) using the Burrows Wheeler Alignment tool [20] v0.7.15-r1140. Once aligned, low mapping quality (mapQ lower than 60) and those sequences flagged as secondary alignments, unproperly paired and/or unmapped sequences in BAM file were removed using SAMtools suite v.1.9 [21]. Additionally, PCR duplicates were removed using the MarkDuplicates tool from Picard suite v.2.17 [22].

CNV analysis was performed using an algorithm developed by Sistemas Genomicos SL divided into six main steps: (i) depth coverage calculation from panel regions, (ii) control samples selection based on coverage correlation (R-squared equal or higher than 0.9 were expected for comparable samples), (iii) depth coverage normalization by GC content and library size, (iv) baseline calculation from normalized coverages of control samples, (v) log2 ratio between normalized coverage of case and the built-in baseline, (v) a circular binary segmentation step to identify supported segmentation breakpoints, and (vi) copy number estimation of segmented regions. If copy number estimation of segmented region is greater or lower than duplication or deletion cutoffs (2.5 and 1.5, respectively) a CNV is reported. Interpretation of the results was performed with the Genesystems platform through its pipeline for CNV analysis (Sistemas Genómicos, Valencia, Spain).

Structural variants were identified and annotated using the Manta algorithm [23] and SnpEff v.4.3 [24].

### 2.3. Conventional Cytogenetic Analysis and MLPA

Routine chromosome analysis could be performed in 121 of the 138 patients included in this study. Unstimulated culture of bone marrow cells was carried out for 24 hours. Preparations were GTL-banded with trypsin and Leishman’s stain and at least 20 metaphases from each culture were analyzed. Karyotypes were described according to the International System for Human Cytogenetic Nomenclature (ISCN 2016) [25]. FISH analysis for *KMT2A* (break apart probe), *MECOM/RPN1* rearrangements (dual color/dual fusion probe) and for *TP53* deletion was performed in all AML patients as clinical routine testing. In all those cases with absence or suboptimal cell culture growth (less than 15 metaphases without alterations), FISH probes for del(5q)/monosomy 5, del(7q)/monosomy 7, del(20q) and aneusomies of chromosome 8 were added as routine analysis. FISH analysis was performed as indicated by the manufacturer’s protocol (Vysis, Abbott Molecular, Chicago, IL, USA). A total of 200 nuclei were analyzed considering 7% as positivity cut-off for single locus and break-apart probes, and 2.3% for dual color/dual fusion probes.

For validation of some specific CNV, *ETV6/RUNX1* (dual color/dual fusion probe), *CBFB* (break apart), P16/cep9 and cep16 FISH probes were also used. Multiplex ligation-dependent probe amplification (MLPA) was performed using SALSA MLPA P335 ALL-IKZF1, P437 Familial MDS-AML and P414 MDS probe mixes (MRC-Holland, Amsterdam, Netherlands) in order to validate CNVs not detected by cytogenetic techniques. MLPA analysis software Coffalyser was used for data analysis.

## 3. Results

### 3.1. Sequencing Coverage and Quality

Sequencing analysis generated an average of seven million reads reaching a median target coverage of 300× per sample when excluding duplicates. The percentage of high-quality mapped reads was 97% and 55% after excluding duplicates. The table with coverage of each region of the NGS panel is shown in Table S2.

### 3.2. Detection of Frequent Alterations by Cytogenetics and/or NGS

**Figure 1:**
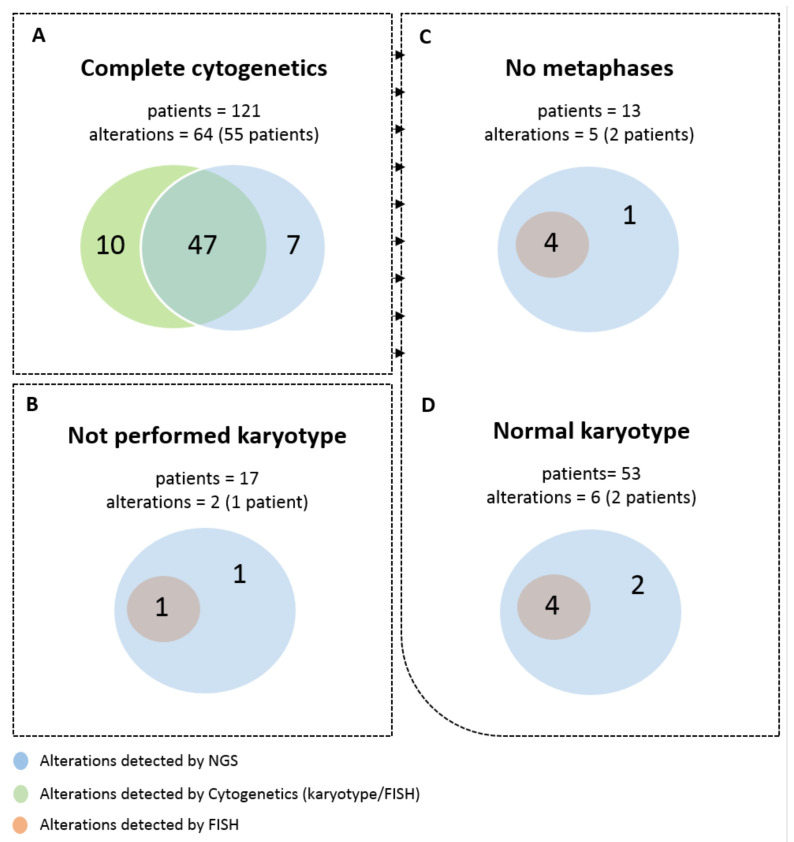
Total number of frequent alterations observed by both NGS panel and cytogenetics. (**A**) All patients with complete cytogenetic analysis performed. (**B**) Patients without karyotype study. (**C**) Patients in which the cytogenetic analysis did not render metaphases to analyze. (**D**) and patients with normal karyotype.

Of the 121 cases with complete cytogenetic study, 64 frequent alterations were detected, 10 (15.6%) of which were detected only by cytogenetics and 7 (10.9%) only by NGS (Figure 1A). In the 17 patients in which karyotype was not performed, two frequent alterations were observed by NGS, one observed also by FISH (Figure 1B). As a total, 66 frequent alterations included in the NGS panel were detected by cytogenetics and/or NGS: 48 (72.7%) by both techniques, 10 (15.1%) only by cytogenetics and eight (12.1%) only by NGS. The most common were those involving chromosome 7 (24%, 16/66) followed by alterations in chromosomes 8 (18%, 12/66), 5 (17%, 11/66), 12 (12%, 8/66) and 20 (11%, 7/66).

Of the 66 frequent alterations detected, eight were myeloid rearrangements, two inv(16), two t(15;17), two t(8;21), one t(9;22) and one t(10;11). NGS detected all of them except for one inv(16), whereas cytogenetics detected all of them except for t(10;11).

Out of the 10 alterations only observed by conventional methods, the most frequent was trisomy 8 (Table 1). In patient number (PN) 3 only FISH analysis performed in isolated CD34+ cells allowed the detection of monosomy 7. This approach overrated the presence of the alteration, which was undetectable when analyzing the whole unfractionated sample either by FISH or by NGS. In PN 12, 14, 17, 46, 56, 64, 72 and 101, alterations represented less than a half of the cells analyzed by karyotype. In light of these results, an additional FISH analysis in a non-cultured sample was performed, in order to evaluate whether the culture would be overestimating the abnormal clone. In six of the nine cases the percentage of the alteration observed by FISH was lower or in the limit of the cut-off established. Moreover, FISH performed in cultured samples showed similar results from those observed in non-cultured samples (Table 1). On the other hand, eight alterations were detected by NGS and not by cytogenetics, mainly deletion of 12p (Table 2). It is worth mentioning that NGS analysis was able to detect the partner implicated in *KMT2A* rearrangement (*KMT2A*/*MLLT10*), whereas cytogenetics only detected *KMT2A* rearrangement by a break-apart FISH probe.

Of the 121 patients with a complete cytogenetic study, no metaphases were obtained in 13, in which a FISH test was performed. In two of the 13 patients, NGS was able to detect five frequent alterations, one of them only detected by NGS (Figure 1C). Of the patients with an optimal karyotype study, 53 presented normal karyotype, three of which showed frequent alterations by FISH and/or NGS, two of them detected only by NGS (Figure 1D).

### 3.3. Secondary CNV Detected by NGS

**Figure 2:**
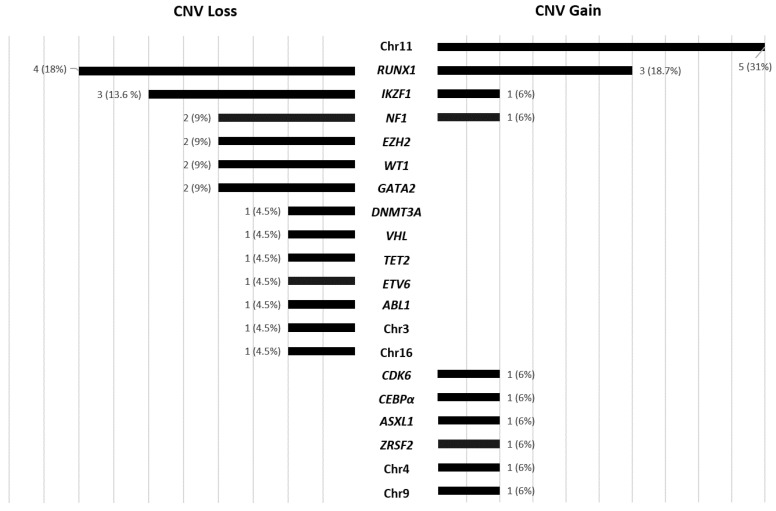
Secondary CNV gains and losses involving genes included in the panel for mutational status analysis: chr11, chr3, chr16, chr4 and chr9 represented CNV gain or loss in all the genes included for these chromosomes.

Aside from frequent alterations, we also observed 38 CNVs (29 patients), 16 gains and 22 losses within some of the genes included in the panel for mutational status analysis (Figure 2). All of them but seven were confirmed by MLPA or cytogenetics (Table S3). CNV losses were more frequent in *RUNX1* (4/22) and *IKZF1* (3/22) genes, as well as *EZH2*, *NF1* and *WT1*, which also presented CNV loss in more than one patient (Figure 2). Genes that showed CNV gains in a higher frequency were *RUNX1* (3/16) and those enclosed in chromosome 11 (5/16), mainly observed by cytogenetics as trisomies of chromosomes 11 and 21 (Table S4).

### 3.4. Secondary CNVs Detected by Karyotype

Other structural and numeric alterations were observed by karyotype analysis, detection of which was not considered in the design of the NGS panel (Table S4). In total, 64 additional cytogenetic alterations were observed in 32 patients. In 13 of these patients, the alterations (*n* = 41) were within a complex karyotype context. In the other 19 patients the observed alterations (*n* = 23) were structural alterations or markers of unknown origin which did not have a real impact on prognosis and management of these patients.

### 3.5. Possible Impact of CNV Detection by NGS in Prognosis Classification

Finally, we analyzed if those frequent alterations detected by NGS analysis could have changed the prognosis in some of the studied cases. Most of the alterations observed only by NGS were del(12p), which was present not as a sole alteration and only changed the prognosis of the disease in PN 23 where the inclusion of an additional alteration changed the disease to unfavorable prognosis. The detection of a del(5q) in PN 47 also changed the prognosis of the disease from intermediate to unfavorable prognosis.

**Figure 3:**
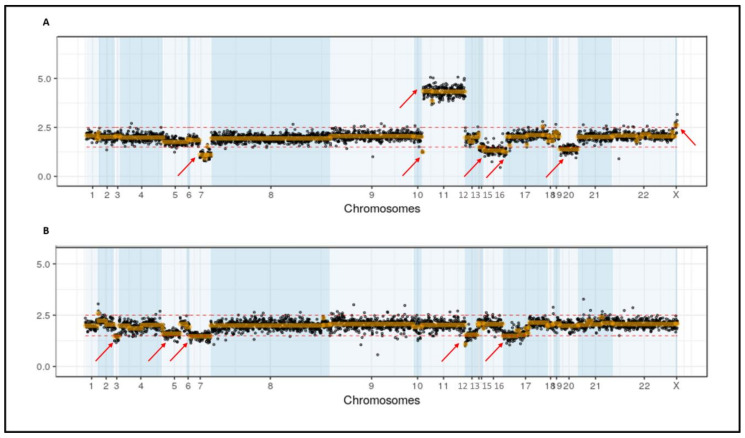
CNV diagram of two patients with more than three CNV alterations: Diagram represented all regions covered sorted by chromosome. CNV gains were considered over 2.5 and CNV losses bellow 1.5. (**A**) CNV diagram of PN 97, who showed a normal karyotype in less than 15 metaphases, which shows seven CNV alterations (arrows), three of them were detected also by routine FISH. (**B**) CNV diagram of PN 60, who did not have an available karyotype, showing five CNV alterations, four of them detected also by routine FISH.

When analyzing the impact of NGS versus only karyotype results, 19 frequent alterations were detected in 12 patients. In five of these patients the alterations were present within a context of an altered karyotype not influencing prognosis. The other six patients were in a context of normal karyotype (3/12) or not available karyotype (3/12). Out of these six patients, four (PN 47, PN 60 and PN 97) would have changed from intermediate to unfavorable prognosis and one (PN 57) from intermediate to favorable prognosis. Remarkably, two of these patients (PN 60 and PN 97) presented more than three CNVs (Figure 3A,B).

## 4. Discussion

In recent years, NGS has emerged as a useful and reliable method for the detection of genetic mutations, such as single nucleotide variants and short insertions and deletions. However, detection of larger mutations, namely structural and numeric chromosomal alterations by NGS, is still not implemented in routine analysis. In most of the studies reported, detection of CNVs was performed by whole genome sequencing [12,14]. Although it allows the detection of CNVs all along the genome, the depth (number of sequences read) of the analysis needed in cancer studies results in a significant increase in the cost of the analysis, which makes it far from being implemented in clinical routine.

Despite karyotype and FISH being the gold standard techniques for detection of chromosomal alterations, the main advantage of NGS analysis versus standard cytogenetic methods is that NGS analysis does not need an optimal cell growth in culture. Moreover, it allows the screening of mutations and chromosomal alterations in a single analysis starting from a DNA sample.

Within this scenario, in this study we report on the usefulness of a commercial NGS panel for the detection of mutations and chromosomal alterations in MN. Mutation analysis of a common cohort was reported previously showing the utility of this NGS panel in the analysis of mutational landscape of MN [26].

When comparing frequent alterations observed by NGS with those obtained by cytogenetics, results were coincident in 36 patients (46 alterations). On the other hand, discrepancies were observed in 18 cases, of which the alteration was observed by cytogenetics and not by NGS in 10 of them, and by NGS and not by cytogenetics in the remaining eight patients. In some of these cases this could be due to the sensitivity of the technique, a parameter that could be optimized by increasing the depth of analysis. Nevertheless, it is notably that the most frequent alteration not detected by NGS was trisomy 8 (six cases). Furthermore, in five of the six cases this alteration was not observed or was in the limit of detection either by FISH analysis on both, cultured or uncultured samples. Although trisomy 8 in MN prognosis has been related to a certain level of mosaicism in the normal population [27,28] and as an isolated alteration does not define the presence of MDS in the lack of other criteria [29], it is also an alteration classified within the intermediate prognosis group for AML and recently as an unfavorable prognosis marker in primary myelofibrosis [30]. Moreover, it has also been reported that in MDS patients presenting myeloproliferative features as well as in CMML patients, carrying trisomy 8 is linked to a worse prognosis [31,32]. Whether proliferation-related alterations have a role in the progression of the disease is something to be studied in the future. However, this observation could be related with the depth of the covered regions and has to be taken into account in the future for a correct detection of this kind of alteration.

On the other hand, eight alterations were detected only by NGS. The most frequent alteration not observed by cytogenetics was del(12p) (6/8). Considering the total of del(12p) detected, the frequency of this alteration goes up to 8% (4/49) in our AML cohort and up to 7.8% (3/38) in MDS patients. These findings are in accordance with Braukle et al., who observed a higher incidence of del(12p) by FISH performed in CD34+ selected cells of MDS patients compared to the results obtained by karyotype (7.4% vs. 1.6%) [33]. This alteration is detected routinely by karyotype and it is reported in approximately 5% of MN, frequently associated with other alterations and complex karyotype. The use of techniques with higher resolution such as NGS could increase the rate of detection of some alterations, such as del(12p), and thus its frequency and possible impact in MN.

Among all the alterations only detected by NGS, a cryptic rearrangement of the *KMT2A* gene was also observed. Rearrangements by NGS are usually analyzed on RNA samples [15,34,35]. Although the sensitivity reached is not enough to use it as a follow-up technique, the detection of rearrangements in seven cases by our custom-panel indicates that DNA designed panels could be a good alternative for the detection of frequent rearrangements at diagnosis of the myeloid disease.

CGH array is the most used methodology for subcytogenetic CNV analysis; however, it is not performed for MN diagnosis as a routine test as it is not mandatory and not always available in all laboratories. Interestingly, while analyzing structural CNV, gains and losses within genes included in the panel for mutational analysis were also observed. The most frequent gains were those affecting *RUNX1* and genes on chromosome 11, five of them corresponding to trisomies of chromosomes 21 and 11 alterations frequently observed in MN [36,37,38], which were also uncovered by karyotype analysis. *RUNX1* was also the gene that presented CNV losses more frequently (4/22), followed by *IKZF1* (3/22). *RUNX1* intragenic deletions as well as point mutations have been described as the cause of familial platelet disorders with propensity to acute myeloid leukemia [39,40], suggesting that loss of *RUNX1* function could be involved in myeloid transformation. Deletions of IKZF1 are frequent and they are classified as a poor prognosis marker in ALL [41]; however, it is described as a rare alteration in MN and within a 7p deletion landscape [42]. In our series, one of the *IKZF1* deletions was observed in ALL and the other two in MDS, suggesting that deletions in *IKZF1* could also be more frequent than reported in MN.

Deletions in *NF1*, *WT1* and *EZH2* were also observed in more than one patient. While NF1 deletions are reported to be frequent in MN and with a similar frequency in AML to the one observed in our series (4%, 2/49) [43,44], deletions in *WT1* and *EZH2* are scarcely reported [45,46,47]. The two deletions observed in *EZH2*, as well as one of those observed in *WT1*, were observed in MPAL patients and the remaining deletion of *WT1* was detected in a T-ALL patient. Cryptic deletion of *EZH2* in MPAL has already been reported, suggesting that it would be a frequent alteration in this disease [47]. Regarding WT1, mutations in this gene are frequently reported usually attached to the loss of the other copy of the gene in T-ALL [48]. The detection of CNV in two of the three cases of ALL exceptionally included in the analysis suggests the need to include CNV detection in lymphoid disease specific NGS panels.

Conventional cytogenetics detected almost all the reported alterations with clinical impact when complemented with FISH analysis; therefore, CNV results only change prognosis classification in two of all the cases analyzed. However, the use of these kinds of panels allowed us to detect mutations and important cytogenetic alterations simultaneously in one single procedure. Furthermore, the detection of secondary CNVs not observed by cytogenetics could be important for prognosis stratification in the future. Losses in some of the analyzed genes such as *RUNX1*, which have remained undetectable due to the low resolution of gold standard techniques, could have an impact in prognosis. It is also remarkable that in one patient with normal karyotype and in one with no metaphases more than three CNVs were detected. Such results could suggest the existence of a complex karyotype in those cases, as three or more frequent alterations were observed in each of them.

This NGS panel has proved to be a valuable complementary technique; however, as it is a small targeted NGS panel, big translocations such as *MECOM*/*GATA1* rearrangement had to be excluded from the design, as breakpoints are extremely variable and located in a wide extension within the two regions. Although our panel was able to detect 46% of the complex karyotypes included in the study, the detection of complex karyotypes was not one of the aims in the design of this panel. Increasing covered regions and depth of the panel could improve the detection of both *MECOM*/*GATA1* rearrangements and complex karyotypes; however, this would mean an increase in the cost of the probe.

Another point to consider is that an early detection of some alterations such as *FLT3* mutations or a complex karyotype is important for the initiation and selection of the most suitable treatment. While NGS panel results are available in 7–10 days, conventional methods such as cytogenetics and PCR could give information in 1–3 days.

For all the previously mentioned points, nowadays NGS targeted panels are not enough to perform a complete diagnosis in MN and should not be considered as a substitutive but as a complementary technique. However, this is a reduced but complete panel which englobes all the genes with risk stratification importance included in the WHO classification for MN and also includes some of the cytogenetic alterations with prognosis value. These kinds of NGS panels are able to complement the mutational status of myeloid-related genes with additional cytogenetic information that could be of considerable value in those cases with a lack of cytogenetic information, either for cases with insufficient cell growth culture or for those without sufficient bone marrow material to complete the cytogenetic analysis.

## 5. Conclusions

Our study shows that NGS represents a reliable complementary source of information for the analysis of CNVs and translocations. Whereas karyotype is able to detect alterations all over the genome, being the unique technique for the detection of complex karyotypes, NGS panels are limited to the detection of alterations within covered regions. Whole genome/exome sequencing could approach the detection of all genome alterations; however, to date these kinds of approaches are not available in all laboratories as clinical routine methods. The inclusion of NGS targeted sequencing panels in the clinical routine allows the obtaining of information about genes related to the disease in order to improve the risk prognosis stratification and detect possible treatment target mutations. Nowadays NGS targeted panels are a great alternative to obtain mutational and CNV information; however, the time of response and not being able to visualize all the genome alterations makes necessary the performance of complementary techniques such as cytogenetics and more rapid tests such as RT-PCRs. The implementation of chromosomic alterations detection in these kinds of panels is useful not only as a confirmation of the alterations observed by cytogenetics but also to detect small deletions or amplifications not observed by standard techniques. Ultimately, in cases with insufficient material to perform cytogenetic studies, the incorporation of cytogenetic alterations in these kinds of panels could improve the diagnosis of the patient.

## Figures and Tables

**Table 1:** Alterations observed only by cytogenetics.

				% of the Alteration by FISH	
PN	Disease	Alteration	Method	Non-Cultured Sample	Cultured Sample
3	MDS	Monosomy 7	FISH *	90% *	NA
12	MDS/MPN	del(12p)	Karyotype (2/20)	3.6%	6.4%
14	MDS	Trisomy 8	Karyotype (7/20)	10.4%	15.2%
17	AML	inv(16)	Karyotype (2/6)	20%	30%
46	AML	Trisomy 8	Karyotype (4/10)	7.2%	9.8%
56	MDS	Trisomy 8	Karyotype (3/20)	9.8%	13.8%
64	AML	Trisomy 8	Karyotype (5/11)	5.4%	5.2%
72	MDS	Trisomy 8	Karyotype (3/13)	18.4%	22.4%
97	MPAL	del(5q)	FISH	20.50%	21%
101	MDS	Trisomy 8	Karyotype (2/20)	5%	7.8%

In those cases where karyotype was available the abnormal clone is represented over the total of metaphases analyzed. PN: Patient number, NA: Not available, MDS: myelodysplastic syndrome, MDS/MPN: myelodisplastic/myeloproliferative neoplasm, AML: acute myeloid leukemia, MPAL: acute leukemia of ambiguous lineage, * FISH analysis was performed over CD34+ isolated cells. Alterations bellow or in the limit of the cutoff of the FISH probe (those observed in less than 10% of the population).

**Table 2:** Alterations only observed by NGS.

PN	Disease	Alteration Detected by NGS	Karyotype	FISH	Method of Validation
23	AML	del(12p)	46,XY,t(1;10)(p36;q11),inv(2)(q31q37)(14)/46,XY(6)	NP	MLPA
36	MPN	Trisomy 19	NP	del(20q)	MLPA
47	MPAL	del(5q)	46,XY(20)	NP	MLPA
49	MDS	del(12p)	46,XY,add(7)(q22)(3)/46,XY(9)	del(7q)	MLPA
55	AML	t(10;11)	47,X,t(Y;15)(q11;p11),+8,inv(12)(q13q15)(19)/46,XY(1)	MLL rearranged	RT-QPCR
60	AML	del(12p)	NM	del(7q),del(5q),del(17p), loss of MECOM/RPN1	MLPA
109	AML	del(12p)	43,XX,−1,del(2)(p11),−3,der(3)t(1;3)(p11;p23),−5,−7, add(11)(p14),add(19)(q13),+mar(cp15)/46,XX(5)	−7, del(5q)	MLPA
110	MDS	del(12p)	46,XX(15)	NP	MLPA
123	MDS	del(12p)	43,XY,del(3)(p13),−5,−7,add(12)(p13),−20, dmin(7)/46,XY(13)	NP	MLPA

PN: Patient number, NP: Not Performed, NM: No metaphases.

## Data Availability

The data presented in this study are available in Supplementary Materials.

## Supplementary Materials

The following are available online at https://www.mdpi.com/article/10.3390/cancers13123001/s1, Figure S1: Flowchart cohort of patients, Table S1: Genes and frequent chromosomal alterations included in the design of the NGS panel, Table S2: Table of coverage, Table S3: Secondary CNV detected by NGS panel, Table S4: Cytogenetic alterations.

## References

[1] Arber D.A., Orazi A., Hasserjian R., Thiele J., Borowitz M.J., Le Beau M.M., Bloomfield C.D., Cazzola M., Vardiman J.W. (2016). The 2016 revision to the World Health Organization classification of myeloid neoplasms and acute leukemia. Blood.

[2] Döhner H., Estey E., Grimwade D., Amadori S., Appelbaum F.R., Büchner T., Dombret H., Ebert B.L., Fenaux P., Larson R.A. (2017). Diagnosis and management of AML in adults: 2017 ELN recommendations from an international expert panel. Blood.

[3] Greenberg P.L., Tuechler H., Schanz J., Sanz G., Garcia-Manero G., Solé F., Bennett J.M., Bowen D., Fenaux P., Dreyfus F. (2012). Revised international prognostic scoring system for myelodysplastic syndromes. Blood.

[4] Rack K.A., van den Berg E., Haferlach C., Beverloo H.B., Costa D., Espinet B., Foot N., Jeffries S., Martin K., O’Connor S. (2019). European recommendations and quality assurance for cytogenomic analysis of haematological neoplasms. Leukemia.

[5] Gonzales P.R., Mikhail F.M. (2017). Diagnostic and Prognostic Utility of Fluorescence In situ Hybridization (FISH) Analysis in Acute Myeloid Leukemia. Curr. Hematol. Malig. Rep..

[6] Kanagal-Shamanna R., Hodge J.C., Tucker T., Shetty S., Yenamandra A., Dixon-McIver A., Bryke C., Huxley E., Lennon P.A., Raca G. (2018). Assessing copy number aberrations and copy neutral loss of heterozygosity across the genome as best practice: An evidence based review of clinical utility from the cancer genomics consortium (CGC) working group for myelodysplastic syndrome, myelodysplastic/myeloproliferative and myeloproliferative neoplasms. Cancer Genet..

[7] Mehrotra M., Luthra R., Ravandi F., Sargent R.L., Barkoh B.A., Abraham R., Mishra B.M., Jeffrey Medeiros L., Patel K.P. (2014). Identification of clinically important chromosomal aberrations in acute myeloid leukemia by array-based comparative genomic hybridization. Leuk. Lymphoma.

[8] Zahir F.R., Marra M.A. (2015). Use of Affymetrix Arrays in the Diagnosis of Gene Copy-Number Variation. Curr. Protoc. Hum. Genet..

[9] Praulich I., Tauscher M., Göhring G., Glaser S., Hofmann W., Feurstein S., Flotho C., Lichter P., Niemeyer C.M., Schlegelberger B. (2010). Clonal heterogeneity in childhood myelodysplastic syndromes-challenge for the detection of chromosomal imbalances by Array-CGH. Genes Chromosom. Cancer.

[10] Rabin K.R., Man T.K., Yu A., Folsom M.R., Zhao Y.J., Rao P.H., Plon S.E., Naeem R.C. (2008). Clinical utility of array comparative genomic hybridization for detection of chromosomal abnormalities in pediatric acute lymphoblastic leukemia. Pediatr. Blood Cancer.

[11] Tallman M.S., Wang E.S., Altman J.K., Appelbaum F.R., Bhatt V.R., Bixby D., Coutre S.E., De Lima M., Fathi A.T., Fiorella M. (2019). Acute Myeloid Leukemia, Version 3.2019, NCCN Clinical Practice Guidelines in Oncology. J. Natl. Compr. Cancer Netw..

[12] Duncavage E.J., Schroeder M.C., O’Laughlin M., Wilson R., MacMillan S., Bohannon A., Kruchowski S., Garza J., Du F., Hughes A.E.O. (2021). Genome Sequencing as an Alternative to Cytogenetic Analysis in Myeloid Cancers. N. Engl. J. Med..

[13] Mack E.K.M., Marquardt A., Langer D., Ross P., Ultsch A., Kiehl M.G., Mack H.I.D., Haferlach T., Neubauer A., Brendel C. (2019). Comprehensive genetic diagnosis of acute myeloid leukemia by next-generation sequencing. Haematologica.

[14] McKerrell T., Moreno T., Ponstingl H., Bolli N., Dias J.M.L., Tischler G., Colonna V., Manasse B., Bench A., Bloxham D. (2016). Development and validation of a comprehensive genomic diagnostic tool for myeloid malignancies. Blood.

[15] Yao R., Yu T., Qing Y., Wang J., Shen Y. (2019). Evaluation of copy number variant detection from panel-based next-generation sequencing data. Mol. Genet. Genomic Med..

[16] Kluk M.J., Lindsley R.C., Aster J.C., Lindeman N.I., Szeto D., Hall D., Kuo F.C. (2016). Validation and Implementation of a Custom Next-Generation Sequencing Clinical Assay for Hematologic Malignancies. J. Mol. Diagn..

[17] Vosberg S., Herold T., Hartmann L., Neumann M., Opatz S., Metzeler K.H., Schneider S., Graf A., Krebs S., Blum H. (2016). Close correlation of copy number aberrations detected by next-generation sequencing with results from routine cytogenetics in acute myeloid leukemia. Genes Chromosom. Cancer.

[18] Neumann M., Vosberg S., Schlee C., Heesch S., Schwartz S., Gökbuget N., Hoelzer D., Graf A., Krebs S., Bartram I. (2015). Mutational spectrum of adult T-ALL. Oncotarget.

[19] Jerchel I.S., Hoogkamer A.Q., Ariës I.M., Steeghs E.M.P., Boer J.M., Besselink N.J.M., Boeree A., Van De Ven C., De Groot-Kruseman H.A., De Haas V. (2018). RAS pathway mutations as a predictive biomarker for treatment adaptation in pediatric B-cell precursor acute lymphoblastic leukemia. Leukemia.

[20] Li H., Durbin R. (2009). Fast and accurate short read alignment with Burrows-Wheeler transform. Bioinformatics.

[21] Li H., Handsaker B., Wysoker A., Fennell T., Ruan J., Homer N., Marth G., Abecasis G., Durbin R., Project G. (2009). The Sequence Alignment/Map format and SAMtools. Bioinform. Appl. Note.

[22] Picard Tools—By Broad Institute. https://broadinstitute.github.io/picard/.

[23] Chen X., Schulz-Trieglaff O., Shaw R., Barnes B., Schlesinger F., Källberg M., Cox A.J., Kruglyak S., Saunders C.T. (2016). Manta: Rapid detection of structural variants and indels for germline and cancer sequencing applications. Bioinformatics.

[24] Cingolani P., Platts A., Wang L.L., Coon M., Nguyen T., Wang L., Land S.J., Lu X., Ruden D.M. (2012). A program for annotating and predicting the effects of single nucleotide polymorphisms, SnpEff: SNPs in the genome of Drosophila melanogaster strain w1118; iso-2; iso-3. Fly.

[25] McGowan-Jordan J., Simons A., Schmid M., International Standing Committee on Human Cytogenomic Nomenclature (2016). ISCN: An International System for Human Cytogenomic Nomenclature.

[26] Carbonell D., Suárez-González J., Chicano M., Andrés-Zayas C., Triviño J.C., Rodríguez-Macías G., Bastos-Oreiro M., Font P., Ballesteros M., Muñiz P. (2019). Next-Generation Sequencing Improves Diagnosis, Prognosis and Clinical Management of Myeloid Neoplasms. Cancers.

[27] Maserati E., Aprili F., Vinante F., Locatelli F., Amendola G., Zatterale A., Milone G., Minelli A., Bernardi F., Lo Curto F. (2002). Trisomy 8 in myelodysplasia and acute leukemia is constitutional in 15–20% of cases. Genes Chromosom. Cancer.

[28] Saumell S., Solé F., Arenillas L., Montoro J., Valcárcel D., Pedro C., Sanzo C., Luño E., Giménez T., Arnan M. (2015). Trisomy 8, a cytogenetic abnormality in myelodysplastic syndromes, is constitutional or not?. PLoS ONE.

[29] Swerdlow S.H., Campo E., Harris N.L., Jaffe E.S., Pileri S.A., Stein H., Thiele J. (2017). WHO Classification of Tumours of Haematopoietic and Lymphoid Tissues.

[30] Tefferi A., Nicolosi M., Mudireddy M., Lasho T.L., Gangat N., Begna K.H., Hanson C.A., Ketterling R.P., Pardanani A. (2018). Revised cytogenetic risk stratification in primary myelofibrosis: Analysis based on 1002 informative patients. Leukemia.

[31] Drevon L., Marceau A., Maarek O., Cuccuini W., Clappier E., Eclache V., Cluzeau T., Richez V., Berkaoui I., Dimicoli-Salazar S. (2018). Myelodysplastic syndrome (MDS) with isolated trisomy 8: A type of MDS frequently associated with myeloproliferative features? A report by the Groupe Francophone des Myélodysplasies. Br. J. Haematol..

[32] Such E., Cervera J., Costa D., Solé F., Vallespí T., Luño E., Collado R., Calasanz M.J., Hernández-Rivas J.M., Cigudosa J.C. (2011). Cytogenetic risk stratification in chronic myelomonocytic leukemia. Haematologica.

[33] Braulke F., Müller-Thomas C., Götze K., Platzbecker U., Germing U., Hofmann W.-K., Giagounidis A.A.N., Lübbert M., Greenberg P.L., Bennett J.M. (2015). Frequency of del(12p) is commonly underestimated in myelodysplastic syndromes: Results from a German diagnostic study in comparison with an international control group. Genes Chromosom. Cancer.

[34] Afrin S., Zhang C.R.C., Meyer C., Stinson C.L., Pham T., Bruxner T.J.C., Venn N.C., Trahair T.N., Sutton R., Marschalek R. (2018). Targeted Next-Generation Sequencing for Detecting MLL Gene Fusions in Leukemia. Mol. Cancer Res..

[35] Flach J., Shumilov E., Joncourt R., Porret N., Tchinda J., Legros M., Scarpelli I., Hewer E., Novak U., Schoumans J. (2020). Detection of rare reciprocal RUNX1 rearrangements by next-generation sequencing in acute myeloid leukemia. Genes Chromosom. Cancer.

[36] Eisfeld A.-K., Kohlschmidt J., Mrózek K., Blachly J.S., Nicolet D., Kroll K., Orwick S., Carroll A.J., Stone R.M., de la Chapelle A. (2016). Adult acute myeloid leukemia with trisomy 11 as the sole abnormality is characterized by the presence of five distinct gene mutations: MLL-PTD, DNMT3A, U2AF1, FLT3-ITD and IDH2. Leukemia.

[37] Heinonen K., MRÓzek K., Lawrence D., Arthur D.C., Pettenati M.J., Stamberg J., Qumsiyeh M.B., Verma R.S., Maccallum J., Schiffer C.A. (1998). Clinical characteristics of patients with *de novo* acute myeloid leukaemia and isolated trisomy 11: A Cancer and Leukemia Group B study. Br. J. Haematol..

[38] Strati P., Daver N., Ravandi F., Pemmaraju N., Pierce S., Garcia-Manero G., Nazha A., Kadia T., Jabbour E., Borthakur G. (2013). Biological and clinical features of trisomy 21 in adult patients with acute myeloid leukemia. Clin. Lymphoma. Myeloma Leuk..

[39] Cavalcante de Andrade Silva M., Krepischi A.C.V., Kulikowski L.D., Zanardo E.A., Nardinelli L., Leal A.M., Costa S.S., Muto N.H., Rocha V., Velloso E.D.R.P. (2018). Deletion of RUNX1 exons 1 and 2 associated with familial platelet disorder with propensity to acute myeloid leukemia. Cancer Genet..

[40] Vormittag-Nocito E., Ni H., Schmidt M.L., Lindgren V. (2019). Thrombocytopenia and Predisposition to Acute Myeloid Leukemia due to Mosaic Ring 21 with Loss of RUNX1: Cytogenetic and Molecular Characterization. Mol. Syndromol..

[41] Mullighan C.G., Su X., Zhang J., Radtke I., Phillips L.A.A., Miller C.B., Ma J., Liu W., Cheng C., Schulman B.A. (2009). Deletion of *IKZF1* and Prognosis in Acute Lymphoblastic Leukemia. N. Engl. J. Med..

[42] Gur H.D., Wang S.A., Tang Z., Hu S., Li S., Medeiros L.J., Tang G. (2017). Clinical significance of isolated del(7p) in myeloid neoplasms. Leuk. Res..

[43] Boudry-Labis E., Roche-Lestienne C., Nibourel O., Boissel N., Terre C., Perot C., Eclache V., Gachard N., Tigaud I., Plessis G. (2013). *Neurofibromatosis-1* gene deletions and mutations in de novo adult acute myeloid leukemia. Am. J. Hematol..

[44] Haferlach C., Grossmann V., Kohlmann A., Schindela S., Kern W., Schnittger S., Haferlach T. (2012). Deletion of the tumor-suppressor gene NF1 occurs in 5% of myeloid malignancies and is accompanied by a mutation in the remaining allele in half of the cases. Leukemia.

[45] King-Underwood L., Pritchard-Jones K., Zimmermann M., Balgobind B.V., Arentsen-Peters S.T.C.J.M., Alders M., Willasch A., Kaspers G.J.L., Trka J., Baruchel A. (2009). Wilms’ Tumor (WT1) Gene Mutations Occur Mainly in Acute Myeloid Leukemia and May Confer Drug Resistance. Blood.

[46] Xiang Z., Abdallah A.-O., Govindarajan R., Mehta P., Emanuel P.D., Papenhausen P., Schichman S.A. (2015). MYC amplification in multiple marker chromosomes and EZH2 microdeletion in a man with acute myeloid leukemia. Cancer Genet..

[47] Ning Y., Slovak M.L., Schultz R.A., Gojo I., Baer M.R. (2014). Cryptic chromosome abnormalities in a patient with mixed phenotype acute leukemia. Leuk. Lymphoma.

[48] Tosello V., Mansour M.R., Barnes K., Paganin M., Sulis M.L., Jenkinson S., Allen C.G., Gale R.E., Linch D.C., Palomero T. (2009). WT1 mutations in T-ALL. Blood.

