# Supplementary material for: Next Generation Cytogenetics in Myeloid Hematological Neoplasms: Detection of CNVs and Translocations
Source: Cancers (Basel). 2021 Jun 15;13(12):3001. doi: 10.3390/cancers13123001 (PMC8232573; doi:10.3390/cancers13123001)
Supplement: Supplementary file 1 [file cancers-13-03001-s001.zip › cancers-1204988_Supplementary_Publish.pdf]

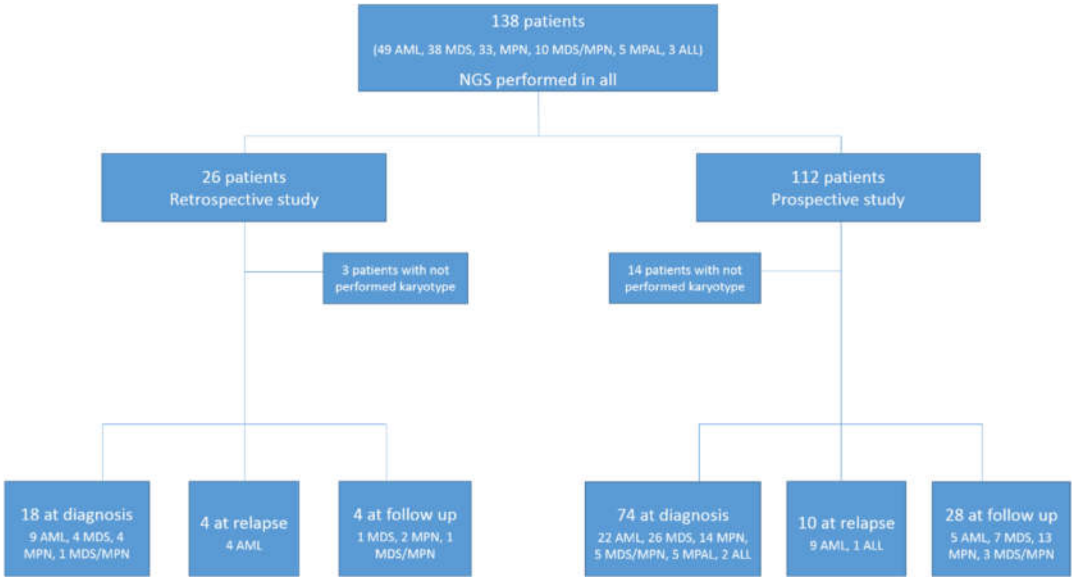

Figure S1. Flowchart cohort of patients.

Table S1. Genes and frequent chromosomal alterations included in the design of the NGS panel

**Frequent chromosomal alterations included in the panel**						
**Numeric/structural alteration**	**Gene**	**Numeric/structural alteration**	**Gene**	**Chromosome**	**Localization**	**Covered region**
-5	**NIPBL**	del(5q)	**APC**	*Chr5*	p13.2	Ex 1–5
	**APC**		**EGR1**		q22.2	Ex 1–4
	**EGR1**		**TCOF1**		q31.2	Ex 1–2
	**TCOF1**		**ATOX1**		q33.1	Ex 1–5
	**ATOX1**				q33.1	Ex 1–3
-7	**IKZF1**	del(7q)	**CDK6**	*Chr7*	p12.1	Ex 2–8
	**CDK6**		**CYP3A5**		q21.2	Ex 1–5
	**CYP3A5**		**CAV1**		q22.1	Ex 1–5
	**CAV1**		**EZH2**		q31.2	Ex 1–3
	**EZH2**				q36.1	Ex 1–20
+8			**NRG1**	*Chr8*	p12	Ex 1–7
			**PRDM14**		q13.3	Ex 1–7
			**NBN**		q21.3	Ex 1–6
			**MYC**		q24.21	Ex 1–3
			**CYP11B2**		q24.3	Ex 1–5
del(11q)			**HEPN1**	*Chr11*	q24.2	Ex 1
			**ST14**		q24.3	Ex 1–5
del(12p)			**ETV6**	*Chr12*	p13.2	Ex 1–8
			**GRIN2B**		p13.1	Ex 1–5
del(17p)			**TP53**	*Chr17*	p13.1	Ex 2–11
+19			**EPOR**	*Chr19*	p13.2	Ex 1–8
			**CALR**		p13.13	Ex 9
			**CEBPa**		q13.11	Ex 1
del(20q)			**SRC**	*Chr20*	q11.23	Ex 1–5
			**HNF4A**		q13.12	Ex 1–10
Rearrangement			***Gene***	***Chromosome***	***Localization***	***Covered region***
t(1;22)(p13;q13) (*RBM15/MKL1*)			**RBM15**	*Chr1*	p13.3	Int 1–2
del(4q)/rearrangement (*FIP1L1/PDFGRA*)			**FIP1L1**	*Chr4*	q12	Ex 1–4 plus Int 8–11
			**PDGFRA**	*Chr4*	q12	Ex 1–5 plus Int 10–12

t(5;var)(q33.1;var) (*PDFGRb/var*)	**PDGFRB**	*Chr5*	q33.1	Int 9–11
t(6;9)(p23;q34) (*DEK/NUP214*)	**DEK**	*Chr6*	p22.3	Int 1
t(8;9)(p22;p24) (*PCM1/JAK2*)	**PCM1**	*Chr8*	p22	Int 26,28–29,36
	**JAK2**	*Chr9*	p24.1	Ex 8, 12–15
t(8;var)(p11.23;var) (*FGFR1/var*)	**FGFR1**	*Chr8*	p11.23	Int 10
t(8;21)(q22;q22) (*RUNX1T1/RUNX1*)	**RUNX1T1**	*Chr8*	q22.1	Int 1
	**RUNX1**	*Chr21*	q22.12	Ex 2–8 plus Int 5
t(9;22)(q34;q11.2) (*BCR/ABL1*)	**ABL1**	*Chr9*	q34.13	Ex 4–11 plus Int 1,2
	**BCR**	*Chr22*	q11.23	Int 1,13–14
t(11;var)(q23.3;var) (*KMT2A/var*)	**KMT2A**	*Chr11*	q23.3	Ex 1–36 plus Int 6–14, 20–24
t(15;17)(q24.1;q21.2) (*PML/RARa*)	**PML**	*Chr15*	q24.1	Ex 6 plus Int 3,6
	**RARa**	*Chr17*	q21.2	Int 2–3
inv(16)(p13q22) (*MYH11/CBFB*)	**MYH11**	*Chr16*	p13.11	Int 6–7,11,27–28,32
	**CBFB**	*Chr16*	q22.1	Int 5

Genes included for variant and small indels analysis

**Gene**	**Chromosome**	**Localization**	**Covered región**
CSF3R	*Chr1*	p34.3	Ex 6–8, 14–17
MPL		p34.2	Ex 3–6, 10, 12
NRAS		p13.2	Ex 2–3
DNMT3A	*Chr2*	p23.3	Ex 7–23
SF3B1		q33.1	Ex 12–16
IDH1		q33.3	Ex 4
VHL	*Chr3*	p25.3	Ex 2, 3
GATA2		q21.3	Ex 3–7
KIT	*Chr4*	q12	Ex 8,17
TET2		q24	Ex 1–11
NPM1	*Chr5*	q35.1	Ex 12
DDX41		q35.3	Ex 1–17
**IKZF1**	*Chr7*	p12.1	Ex 2–8
**EZH2**		q36.1	Ex 1–20
**JAK2**	*Chr9*	p24.1	Ex 8, 12–15
**ABL1**		q34.13	Ex 4–11 plus Int 1,2
ANKRD26	*Chr10*	p12.1	Ex 1–34 plus 5'UTR
WT1	*Chr11*	p13	Ex 7–9
CBL		q23.3	Ex 4–5, 8–11
**ETV6**	*Chr12*	p13.2	Ex 1–8
ETNK1		p12.1	Ex 3
KRAS		p12.1	Ex 2–5
PTPN11		q24.13	Ex 3–4,13
FLT3	*Chr13*	q12.13	Ex 14,20
IDH2	*Chr15*	q26.1	Ex 4
**TP53**	*Chr17*	p13.1	Ex 2–11
NF1		q11.2	Ex 1–58
SRSF2		q25.1	Ex 1
SETBP1	*Chr18*	q12.3	Ex 4
**EPOR**	*Chr19*	p13.2	Ex 1–8
**CALR**		p13.13	Ex 9
**CEBPA**		q13.11	Ex 1
ASXL1	*Chr20*	q11.21	Ex 12–13
**RUNX1**	*Chr21*	q22.12	Ex 2–8 plus Int 5
ZRSR2	*ChrX*	p22.1	Ex 1–11

Genes and frequent chromosomal alterations included in the design of the NGS panel. Chr: chromosome, Ex: exon, Int: Intron. In bold those genes included for structural and numeric chromosome alterations.

Table S3. Secondary CNV detected by NGS panel.

PN	Disease	CNV gene or region	Type of CNV	Method of validation
21	MDS/MPN	*GATA2* Exons 5–7	Loss	MLPA
23	AML	*DNMT3A* Exons 7–23	Loss	NA
27	AML	*RUNX1* Exons 3–8	Gain	Karyotype
34	MDS/MPN	*RUNX1* Exons 3–8	Loss	FISH
38	AML	*RUNX1* Exon 8	Loss	MLPA
40	AML	chr11: All regions covered	Gain	Karyotype
41	MDS	*CDK6* Exons 1–5	Gain	MLPA
		CEBPA Exon 1	Gain	MLPA
42	MPAL	*RUNX1* Exons 3–5	Loss	MLPA
47	MPAL	*EZH2* Exons 2–19	Loss	MLPA
49	MDS	*IKZF1* Exons 2–3	Loss	MLPA
50	MPD	*ABL1* Int 1	Loss	FISH
54	AML	chr11: All regions covered	Gain	MLPA
60	AML	chr3: All regions covered	Loss	FISH
68	ALL	*IKZF1* Exons 4–7	Loss	MLPA
		ETV6 Exons 2–3	Loss	MLPA
69	MDS	*IKZF1* Exons 2–8	Loss	Karyotype/MLPA
		RUNX1 Exons 7–8	Loss	MLPA
76	AML	chr11: All regions covered	Gain	Karyotype
77	AML	*IKZF1* Ex 2–8	Gain	MLPA
78	MPD	chr9: All regions covered	Gain	FISH
85	MDS	*RUNX1* Exons 3–8	Gain	Karyotype
89	AML	*NF1* Exon 58	Gain	NA
95	AML	*NF1* All regions covered	Loss	MLPA
97	MPAL	*WT1* Exons 7–9	Loss	NA
		chr11: 11q23.3–11q24.3	Gain	FISH
		chr16: All regions covered	Loss	FISH
		ZRSR2 Exons 1–11	Gain	NA
108	MPAL	chr4: All regions covered	Gain	Karyotype
109	AML	*GATA2* Exons 3–7	Loss	Karyotype/MLPA
		NF1 All regions covered	Loss	MLPA
111	MDS	*RUNX1* Exons 3–8	Gain	Karyotype
122	ALL-T	*WT1* Exons 7–9	Loss	NA
123	MDS	*VHL* Exons 2–3	Loss	Karyotype
		ASXL1 Exons 11–12	Gain	NA
125	AML	*TET2* Exons 1–11	Loss	NA
134	MPAL	*EZH2* Exons 1–20	Loss	MLPA
		chr11: All regions covered	Gain	MLPA

CNV gains and losses detected in some of the other panel gene enclosed for mutational analysis are listed. These alterations were validated by other techniques when possible. PN: Patient number, NA: Not available.
